# Large-Scale Screening of Asymptomatic Persons for SARS-CoV-2 Variants of Concern and Gamma Takeover, Brazil

**DOI:** 10.3201/eid2712.211326

**Published:** 2021-12

**Authors:** Douglas Adamoski, Jaqueline Carvalho de Oliveira, Ana Claudia Bonatto, Roseli Wassem, Meri Bordignon Nogueira, Sonia Mara Raboni, Edvaldo da Silva Trindade, Emanuel Maltempi de Souza, Daniela Fiori Gradia, SCB-UFPR COVID-19 team,

**Affiliations:** Brazilian Biosciences National Laboratory, Brazilian Center for Research in Energy and Materials, Campinas, Brazil (D. Adamoski);; Universidade Federal do Paraná, Curitiba, Brazil (D. Adamoski, J. Carvalho de Oliveira, A.C. Bonatto, R. Wassem, M.B. Nogueira, S.M. Raboni, E.S. Trindade, E.M. de Souza, D.F. Gradia)

**Keywords:** environment and public health, diagnosis, diagnostic techniques and procedures, COVID-19, coronavirus disease, SARS-CoV-2, severe acute respiratory syndrome coronavirus 2, viruses, respiratory infections, zoonoses, Brazil

## Abstract

We performed a large-scale severe acute respiratory syndrome coronavirus 2 screening campaign using 2 PCR-based approaches, coupled with variant genotyping, aiming to provide a safer environment for employees of Federal University in Curitiba, Brazil. We observed the rapid spread of the Gamma variant of concern, which replaced other variants in <3 months.

Ongoing screening for active severe acute respiratory syndrome coronavirus 2 (SARS-CoV-2) infections, coupled with contact tracing, can efficiently reduce viral transmission within the community (*1*). However, as new and more transmissible variants emerge, an increased number of cases can be observed across affected regions. This increase demands immediate action, such as consistent, uninterrupted genomic surveillance for regular evaluation of vaccination approaches (*2*), to curb virus spread.

Brazil has reaped the consequences of lack of genomic surveillance in a context of high seroprevalence. The variant of concern (VOC) Gamma (B.1.1.28/P.1) emerged in the city of Manaus, Brazil, at a time when three quarters of the population had tested positive for antibodies; it proceeded to spread across the city (*3*). The Gamma variant was able to replace its predecessor, the variant of interest (VOI) B.1.1.28/P.2, previously the dominant lineage in the region (*4*).

Routine SARS-CoV-2 mass screening programs of asymptomatic persons and follow-up genotyping of samples are necessary measures to control the number of cases and prevent further infection surges (*5*,*6*). Simplified approaches, such as multiplex quantitative PCR, provide a feasible, cost-effective way to discriminate samples and prioritize whole-genome sequencing efforts (*5*,*7*). To provide a safer environment for the university community, we performed a large-scale screening for SARS-CoV-2 infections and VOCs in the Federal University of Paraná (Curitiba, Brazil) community. The study was approved by the University Research Ethics Committee (approval no. CAAE: 31687620.2.0000.0096).

## The Study

During October 10, 2020–May 24, 2021, asymptomatic and mildly symptomatic persons within the community of the Federal University of Paraná were called for voluntary participation through social media and email. Eligible participants were members of the academic community (students, technicians, professors, or outsourced employees) or their relatives (grandparents, parents, siblings, or children) or household members. Saliva samples were self-collected by using an individually wrapped plastic drinking straw, transferred to a prelabeled 2.0 mL microtube, and stored at 4°C. Samples were transported to the laboratory in <1 h; total turnaround time to results was <48 h.

Samples were homogenized and allowed to settle for 30 min or centrifuged for 2 min (2,000 × *g*). A quantity of 200 µL from each specimen was then pooled (*8*) in groups of 5. We performed RNA extraction by using an automated magnetic EXTRACTA–RNA and DNA Viral kit (Loccus Biotecnologia, https://loccus.com.br). We performed amplification in 2 ways: on a QuantStudio5 instrument (Thermo Fisher Scientific, https://www.thermofisher.com) using AllPlex nCov-2019 reverse transcription PCR Master Mix Kit (SeeGene, https://www.seegene.com) (*1*) or Molecular SARS-CoV-2 EDx (Bio-Manguinhos/FioCruz, https://www.bio.fiocruz.br) (*2*). If the pool rendered a positive result, we reprocessed samples individually.

We further evaluated positive samples by using 2 probe-based genotyping systems to detect VOCs. The first one was the Vogels et al. (*7*) multiplex approach to detect Spike Δ69–70 and Orf1a Δ3675–3677 deletions as an outcome for distinguishing Alpha, Beta, or Gamma and wild-type or other lineages (*7*). For this approach, we also included the Centers for Disease Control and Preventon N1 target and defined a cycle threshold (C_t_) of <28 on this particular target to evaluate the gene dropouts.

The second approach involved 3 allelic discrimination TaqMan assays (Thermo Fisher Scientific). The proposed readout was P.1 (K417T, N501Y, and E484K), P.2 (only E484K), B.1.1.7 (only N501Y), B.1.351 (N501Y and E484K, failure for K417T assay), and wild-type or others for the absence of mutated alleles (*9*). The discriminating power of this second assay made it possible to distinguish the B1.1.28/P.2 from the wild-type and the Beta/Gamma variants. We performed both assays by using GoTaq Probe 1-Step reverse transcription quantitative PCR System (Promega, https://www.promega.com) in the same instrument.

**Figure 1:**
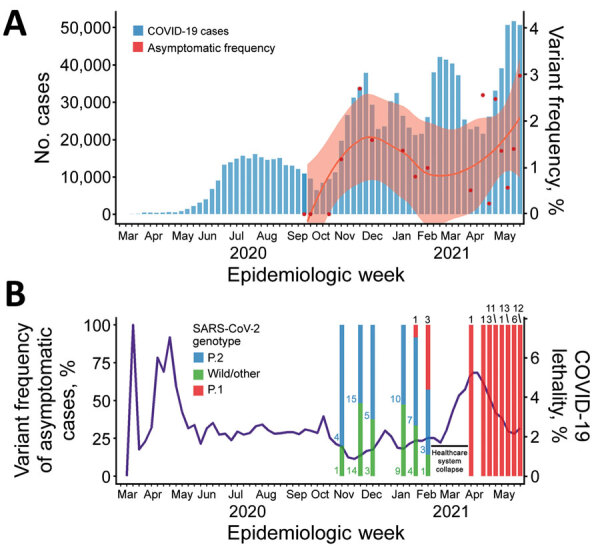
COVID-19 diagnoses, asymptomatic and variant frequency, and lethality, Paraná state, Brazil. A) COVID-19 diagnoses in Paraná and Federal University of Paraná (Curitiba, Brazil) mass testing program positivity rates by epidemiologic week. Blue bars summarize positive cases in Paraná by diagnosis day notified to state surveillance system through February 15, 2021. Red dots represent the fraction of positive cases in all samples from mass screening collection at Federal University of Paraná, smoothed by locally estimated scatterplot smoothing in the red line; pink shading indicates SE from locally estimated scatterplot smoothing fit. B) Overall lethality of COVID-19 in Paraná (purple line) and variant prevalence among asymptomatic and mildly symptomatic cases, by epidemiologic week. Numbers represent the absolute quantity of cases for each variant. Scales for the y-axes differ substantially to underscore patterns but do not permit direct comparisons. COVID-19, coronavirus disease; SARS-CoV-2, severe acute respiratory syndrome coronavirus 2.

A total of 16 collection dates were recorded; 12,558 examinations were processed (Table) from the 7,249 persons who attended because some participants engaged in >1 day of collection. The number of attendees per collection date ranged from 162 to 1,737. The overall prevalence rate was 1.28% (161/12,558). Comparing these numbers to cases in the state of Paraná by the epidemiologic week of diagnosis (Figure 1, panel A), we found prevalence similar to the prevalence rate at the beginning of the state’s second wave of SARS-CoV-2 infections (Figure 1, panel A).

**Table:** Collection dates, engagement, and positivity rates for severe acute respiratory syndrome coronavirus 2 infection, Curitiba, Brazil

Date	Total tested	No. (%) positive
2020 Oct 2	275	0
2020 Oct 19	279	0
2020 Nov 6	510	6 (1.18)
2020 Nov 24	1,265	34 (2.69)
2020 Dec 8	1,070	17 (1.59)
2021 Jan 12	1,692	23 (1.36)
2021 Jan 26	1,737	14 (0.81)
2021 Feb 9	1,615	16 (0.99)
2021 Mar 29	196	1 (0.51)
2021 Apr 12	157	4 (2.55)
2021 Apr 20	872	2 (0.23)
2021 Apr 26	162	4 (2.47)
2021 May 4	884	12 (1.36)
2021 May 10	177	1 (0.56)
2021 May 18	1,431	20 (1.4)
2021 May 24	236	7 (2.97)
Total	12,558	161 (1.28)

**Figure 2:**
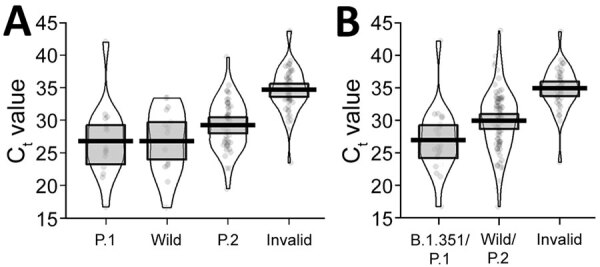
Violin plots of diagnostic C_t_ values for severe acute respiratory syndrome coronavirus 2–positive cases in Paraná state, Brazil, and detection performance for singleplex (A) and multiplex (B) genotyping methods. Violin plots are made by kernel-smoothed density plots from the actual data (represented by the dots). Horizonal lines within boxes indicate medians; upper and lower box limits indicate interquartile ranges. C_t_, cycle threshold.

We also evaluated all SARS-CoV-2–positive cases by using multiplex and singleplex genotyping approaches (Figure 2). From all 161 positive cases evaluated, the Vogels et al. (*7*) multiplex assay was invalidated in 46 (28.6%) against 50 (31.1%) in a Thermo Fisher 3-assay allelic detection approach because of the high C_t_ values. Comparing the original C_t_ value of detection, performance depreciated in samples with C_t_ >30 (Figure 2, panel B), as stated in the Thermo Fisher manual. Nevertheless, all genotyped cases were concordant between the 2 assays, considering that the Vogels et al. (*7*) assay alone does not discriminate between wild-type and B1.1.28/P.2 VOI.

Detection of the Gamma variant occurred on January 21, 2021, <2 weeks after the collapse of the healthcare system in Manaus. Prevalence of the Gamma variant was 9.1% on this date and increased to 42.9% 2 weeks later (Figure 1, panel B). A possible explanation for this scenario is the increased transmission of this variant in >20% compared with the wild-type transmission rate (*10*), which is supported by the observation of a reproduction number of 1.5 in Paraná (http://shiny.leg.ufpr.br/elias/covid19time) in the weeks before the death peak, the highest reproduction number observed during the pandemic. This increase in cases could be correlated with the subsequent collapse of the Curitiba city healthcare system and a surge of coronavirus disease deaths in the Paraná state, reaching values >5% in the subsequent weeks. A similar scenario was also observed in Manaus, the origin of the Gamma variant; both SARS-CoV-2 cases and excess of burials in the city reached their highest levels during the pandemic to that point (*4*). Two factors could explain those observed surges: increased lethality of the Gamma variant—which is not yet defined (*10*)—and the actual collapse of healthcare systems, leading to poorer patient support. When testing activities resumed after the healthcare collapse, all cases became Gamma variant, completely displacing B1.1.28/P.2 VOI and wild-type cases in 3 months.

## Conclusions

Analysis of saliva in pools as described in this study offers an inexpensive and easy-to-implement asymptomatic screening strategy. Thus, given the high rates of SARS-CoV-2 transmission, the risk for asymptomatic coronavirus disease spread and the importance of social distancing should continue to be stressed to the public until the vaccine is viable for large-scale application. Our mass testing program was intended to be accessible (every test was free-of-charge for the participant), reliable (all participants received their results and positive persons had a follow-up opportunity), and aimed to reach all social strata within the academic community (from professors to outsourced employees, which consisted mainly of socially, economically, and ethnically vulnerable groups), which are key characteristics of a strong mass testing system (*11*).

We found that both multiplex PCR and singleplex PCR approaches were valuable tools to evaluate the proportion of variants within genomic surveillance and were faster and less expensive than whole-genome sequencing approaches. Although those methods do not serve as substitutes for whole-genome sequencing, they could be an essential method to screen and select samples for further variant classification. Nevertheless, those approaches could demonstrate rapid spread of new variants and predict surges of SARS-CoV-2 infections, acting as a lighthouse for far-reaching public health decisions.

This article was preprinted at https://www.medrxiv.org/content/10.1101/2021.06.18.21258649v1.
